# The association between ankylosing spondylitis and the risk of venous thromboembolism: a meta-analysis

**DOI:** 10.3389/fimmu.2025.1670965

**Published:** 2025-12-09

**Authors:** Quan Hu, Mei Mei, Ruihan Liu, Qiang Zhang

**Affiliations:** 1 Department of Nephrology, The Shapingba Hospital, Chongqing University(People’s Hospital of Shapingba District, Chongqing), Chongqing, China; 2 Department of Scientific Research, The Shapingba Hospital, Chongqing University(People’s Hospital of Shapingba District, Chongqing), Chongqing, China

**Keywords:** ankylosing spondylitis, venous thromboembolism, meta-analysis, systematic review, risk

## Abstract

**Background:**

Autoimmune diseases are frequently associated with an increased risk of venous thromboembolism (VTE). However, the relationship between ankylosing spondylitis (AS) and VTE remains unclear. This meta-analysis was conducted to evaluate the association between AS and VTE risk, with the aim of providing evidence-based insights to guide clinical practice.

**Method:**

We searched PubMed, Embase, the Cochrane Library, and Web of Science for studies published from database inception until June 2025 that compared VTE risk in patients with AS. Two researchers independently screened the literature based on predefined inclusion and exclusion criteria. The risk of bias in included studies was assessed using the Risk of Bias in Non-randomized Studies of Interventions (ROBINS-I) tool. Adjusted hazard ratios (HRs) and 95% confidence intervals (CIs) were pooled to estimate effect sizes. Subgroup analyses were performed according to the type of thrombosis event. Sensitivity analysis was conducted by sequentially excluding each study. Publication bias was preliminarily assessed using funnel plots. The overall quality of evidence was evaluated using the Grading of Recommendations Assessment, Development and Evaluation (GRADE) framework. All analyses were performed using RevMan 5.3 software. The study protocol was registered retrospectively in PROSPERO (Registration number: CRD420251107387).

**Results:**

Six studies involving 601,585 patients were included. The meta-analysis indicated that AS was associated with a significantly higher risk of VTE (HR = 1.47, 95%CI(1.22,1.77), P < 0.0001; evidence quality: Very low). Subgroup analyses revealed that AS was significantly associated with an increased risk of overall VTE, deep vein thrombosis (DVT), and pulmonary embolism (PE) (VTE: HR = 1.57, 95%CI(1.26, 1.96), P < 0.0001; DVT: HR = 1.62, 95%CI(1.16, 2.26), P = 0.005; PE: HR = 1.24, 95%CI(1.06, 1.45), P = 0.008).

**Conclusion:**

Current evidence indicates that AS may elevate the risk of VTE. Further mechanistic studies are needed to better interpret these findings derived from observational data.

**Systematic Review Registration:**

https://www.crd.york.ac.uk/PROSPERO/, identifier CRD420251107387.

## 1 Introduction

Ankylosing spondylitis (AS) is a chronic inflammatory autoimmune disease that primarily invades the sacroiliac joints, spine, paraspinal soft tissues, and peripheral joints (1, 2). It occurs more frequently in young and middle-aged adults, with a male-to-female incidence radio of approximately 2:1 (3). The clinical manifestations include spinal pain and stiffness, as well as extra-spinal features such as uveitis, inflammatory bowel disease and psoriasis, all of which contribute to a substantial economic and psychological burden on patients (4).

Recent retrospective studies have indicated a significantly elevated risk of venous thromboembolism (VTE) in patients with AS (5–10). VTE is a common condition characterized by the formation of venous blood clots, leading to vascular obstruction. It encompasses two main types: pulmonary embolism (PE) and deep vein thrombosis (DVT) (11). In the United States, there are an estimated 260,000 new cases of VTE diagnosed annually (12). A national discharge survey conducted between 2007 and 2009 reported approximately 548,000 annual hospitalizations related to VTE among U.S. adults, including 349,000 cases of DVT and 278,000 cases of PE (13). One retrospective study of 214,901 American patients initially diagnosed with DVT or PE found that about 4% were readmitted for recurrent PE (8,217 cases) or DVT (9,138 cases), with over half of readmissions occurring within 30 days after the initial DVT diagnosis (14). In China, the annual incidence of VTE increased significantly from 28.1 per 100,000 in 2004 to 48.3 per 100,000 in 2016 (15), while hospitalization rates rose from 3.2 to 17.5 per 100,000 during the same period, reflecting a consistent upward trend in both incidence and hospitalization (16).

The pathophysiology of VTE involves Virchow’s triad: venous stasis, hypercoagulability, and endothelial injury (17). Immune dysregulation can trigger systemic inflammation, leading to the release of inflammatory cytokines that promote endothelial dysfunction and a hypercoagulable state. Inflammation may further facilitate thrombosis by impairing fibrinolysis and altering the balance between procoagulant and anticoagulant factors (18, 19). Several immune-mediated diseases—such as systemic lupus erythematosus, antiphospholipid syndrome, rheumatoid arthritis, Sjögren’s syndrome, inflammatory myopathy, systemic sclerosis, vasculitis, Behçet’s disease, and inflammatory bowel disease—have been associated with an increased risk of VTE (20). A retrospective study involving 182,431 patients revealed that the incidence of thromboembolic events in immune-mediated diseases was 1.49 times higher than in non-immune-mediated conditions (21). Moreover, the mortality rate is significantly higher in patients with autoimmune diseases who develop VTE.

Currently, evidence regarding the risk of VTE in AS remains limited and the association between AS and VTE is not fully established. This meta-analysis aims to synthesize existing clinical studies evaluating this relationship, with the goal of providing updated evidence to support early prevention and improve clinical management of VTE in patients with AS.

## 2 Methods

This meta-analysis followed the Preferred Reporting Items for Systematic Reviews and Meta-Analyses (PRISMA 2020) guidelines (22–24). The study protocol was registered retrospectively in PROSPERO (Registration number: CRD420251107387).

### 2.1 Search strategy

Relevant studies on the association between AS and VTE risk were identified through searches in PubMed, Embase, Web of Science, and the Cochrane Library from database inception to June 2025. Search terms included “spondylitis, ankylosing,” “venous thrombosis,” “pulmonary embolism,” and related keywords, combining Medical Subject Headings (MeSH) with free words. We also manually screened reference lists of included articles for additional relevant publications. The complete search strategy is provided in **Supplementary Table S1**.

### 2.2 Study selection and eligibility criteria

Two reviewers (Q.H. and M.M.) independently screened titles and abstracts using the EndNote X9. Studies were included if they: (1) enrolled patients diagnosed with AS based on International Classification of Diseases (ICD) codes; (2) compared VTE risk between AS patients and healthy or non-AS controls; (3) reported adjusted effect estimates (HR, RR, or OR) with 95% CIs for VTE; and (4) were cohort or case-control studies published in English with a mean or median follow-up of at least one year. We excluded duplicate publications, reviews, case reports, conference abstracts, and studies with incomplete or unavailable data. Disagreements during screening were resolved through discussion, with a senior investigator (Q.Z.) making final decisions when consensus was not reached.

### 2.3 Data extraction

Literature was managed using EndNote X9. Two reviewers (Q.H. and M.M.) independently screened studies based on the eligibility criteria and extracted data using a standardized form. Extracted information included first author, publication year, study location, design, participant characteristics, AS definition, follow-up duration, adjusted covariates (e.g., age, sex, comorbidities, medications), effect estimates with 95% CIs, and risk of bias assessment results. Data were compiled and managed in Excel. Discrepancies were resolved through discussion until consensus was reached. If no agreement can be reached, then a senior investigator (Q.Z.) will make the final decision.

### 2.4 Risk of bias assessment

The Risk of Bias in Non-randomized Studies of Interventions(ROBINS-I) tool was used to assess study quality (25). The tool evaluates seven domains: ①Confounding; ②Selection of participants; ③Classification of Exposures; ④Deviations from intended interventions; ⑤Missing data; ⑥Measurement of outcomes; ⑦Selection of reported results. Two reviewers (Q.H. and M.M.) independently rated each domain as low, moderate, or high risk of bias based on the design and analytical approach of the study, and an overall judgment was derived for each study by integrating the assessments across all domains. If no agreement can be reached, then a senior investigator (Q.Z.) will make the final decision.

### 2.5 Quality of evidence assessment

The overall evidence quality was evaluated using the Grading of Recommendations Assessment, Development and Evaluations(GRADE) framework,which classifies evidence into high, moderate, low, or very low quality. Factors considered for downgrading included: ①Risk of Bias; ②Inconsistency; ③Indirectness;④Imprecision; ⑤Publication Bias. Upgrading factors included: large magnitude of effect; a dose-response gradient and the influence of all plausible residual confounding. Two reviewers (Q.H. and M.M.) independently conducted the assessment, and a final grade was assigned based on consensus. If no agreement can be reached, then a senior investigator (Q.Z.) will make the final decision.

### 2.6 Statistical analysis

Meta-analysis was performed using RevMan 5.3. HRs and 95% CIs were converted to log HRs and standard errors (SEs) for pooling. Heterogeneity was assessed using Cochran’s Q test and the *I²* statistic. An *I²* > 50% and *p* ≤ 0.10 indicated substantial heterogeneity, warranting a random-effects model; otherwise, a fixed-effects model was applied. Subgroup analyses were conducted by thrombosis type (VTE, DVT, PE) to explore potential sources of heterogeneity and to evaluate the consistency of the association across different clinical presentations of thrombosis. Sensitivity analysis was performed by sequentially excluding each study. Publication bias was preliminarily assessed using funnel plots, though interpretation was cautious due to the limited number of included studies (n < 10).

## 3 Result

### 3.1 Literature search results

**Figure 1:**
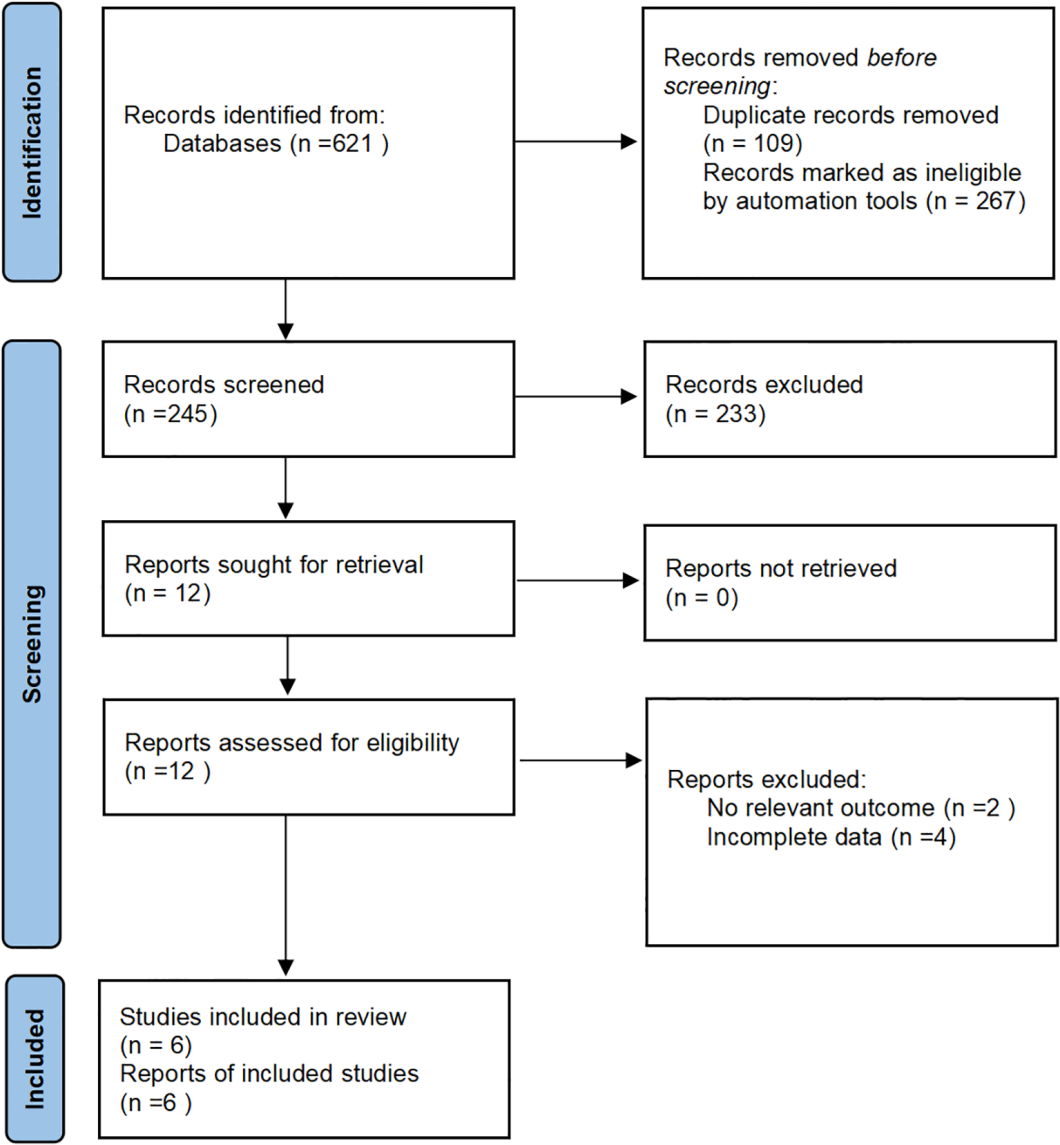
Flowchart of database search and study inclusion.

A total of 621 relevant articles were retrieved from the databases. After removing 109 duplicates using Endnote and excluding reviews, case reports, conference abstracts, and other ineligible study types, 245 articles remained. After reading the titles and abstracts, 217 articles were excluded. Following full-text review based on the inclusion and exclusion criteria, six articles (5–10) were finally included (**Figure 1**).

### 3.2 The baseline characteristics included in the study

The six included studies comprised two from Sweden, and one each from Israel, Canada, the United Kingdom and Denmark. There were five cohort studies (primarily utilizing nationwide or population-based registries) and one case-control study, with a total sample size of 601,585 participants. The study periods ranged from 1964 to 2019, and all studies had a follow-up duration exceeding one year (**Table 1**). Confounding factors adjusted for in each study are summarized in **Supplementary Table S2**. Assessment using the ROBINS-I tool indicated one study had a low risk of bias across all domains. One study was rated as high risk, primarily due to its restriction to inpatients, lack of adjustment for key confounders, and exposure definition based solely on a single inpatient diagnosis, leading to potential exposure misclassification and control group contamination. The remaining four studies were judged as having a moderate risk of bias, mainly arising from potential residual confounding or exposure misclassification (**Supplementary Figure S1**). Although most of the studies exhibit a moderate risk of bias, the nature of the research questions necessitates the use of observational designs. These studies represent the best available evidence at present.

**Table 1:** Basic characteristics of the included studies.

Author (year)	Country	Study design	Study duration	Sample size	Disease definition	Adjustment for covariates	Follow- up(years)	Type of disease
Gendelman et al 2024	Israel	Cohort study	2002- 2019	34181	–	Age,Sex,BMI, Ethnicity, Smoking history, Economic status	7.35 (mean)	PE
Aviña-Zubieta et al., 2019	Canada	Cohort study	1996- 2012	79090	ICD-9, ICD-10	Age,Sex,Drugs,Number of outpatient visits	6.2 (mean)	VTE,PE,DVT
Bengtsson et al 2017	Sweden	Cohort study	2001- 2012	272883	ICD-8,ICD-9,ICD-10	Age,Sex	5.6 (mean)	VTE
Ramagopalan et al 2011	England	Cohort study	1999- 2008	44002	ICD-9	Age,Sex,Time, District of residence, Economic status	10	VTE
Johannesdottir et al., 2012	Danmark	Case- control study	1999- 2009	161931	ICD-8,ICD-9,ICD-10	Age,Sex, District of residence, Comorbidity, Drugs, Trauma/surgical history, Economic status	>1	VTE
Zöller et al 2012	Sweden	Cohort study	1964- 2008	9498	ICD-7,ICD-8,ICD-9,ICD-10	Age,Sex,Time, Comorbidity	>10	PE

ICD, international classification of diseases;BMI,body mass index;VTE, venous thromboembolism; DVT, deep venous thrombosis; PE, pulmonary embolism.

### 3.3 Meta-analysis

**Figure 2:**
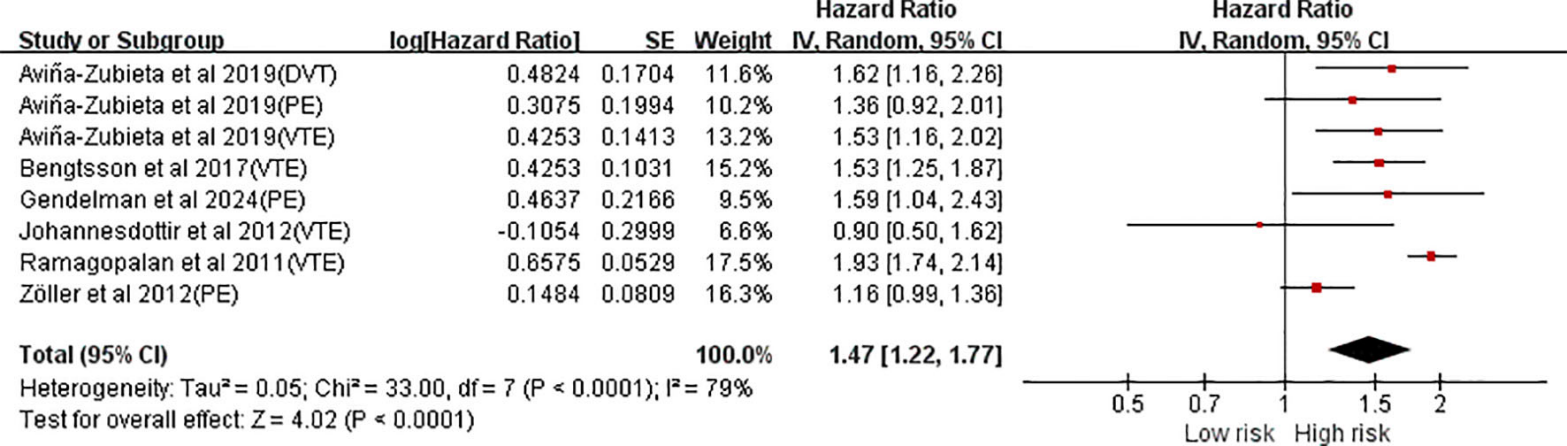
Forest plot of the risk of venous thromboembolism in ankylosing spondylitis.

Six studies, comprising eight independent data reports, assessed the association between AS and VTE risk. Substantial heterogeneity was observed (*I^2^* = 79%, *P* < 0.0001). The meta-analysis showed that AS was associated with a significantly increased risk of VTE (HR = 1.47, 95%CI(1.22, 1.77), *P* < 0.0001) (**Figure 2**).

### 3.4 Subgroup analysis

**Figure 3:**
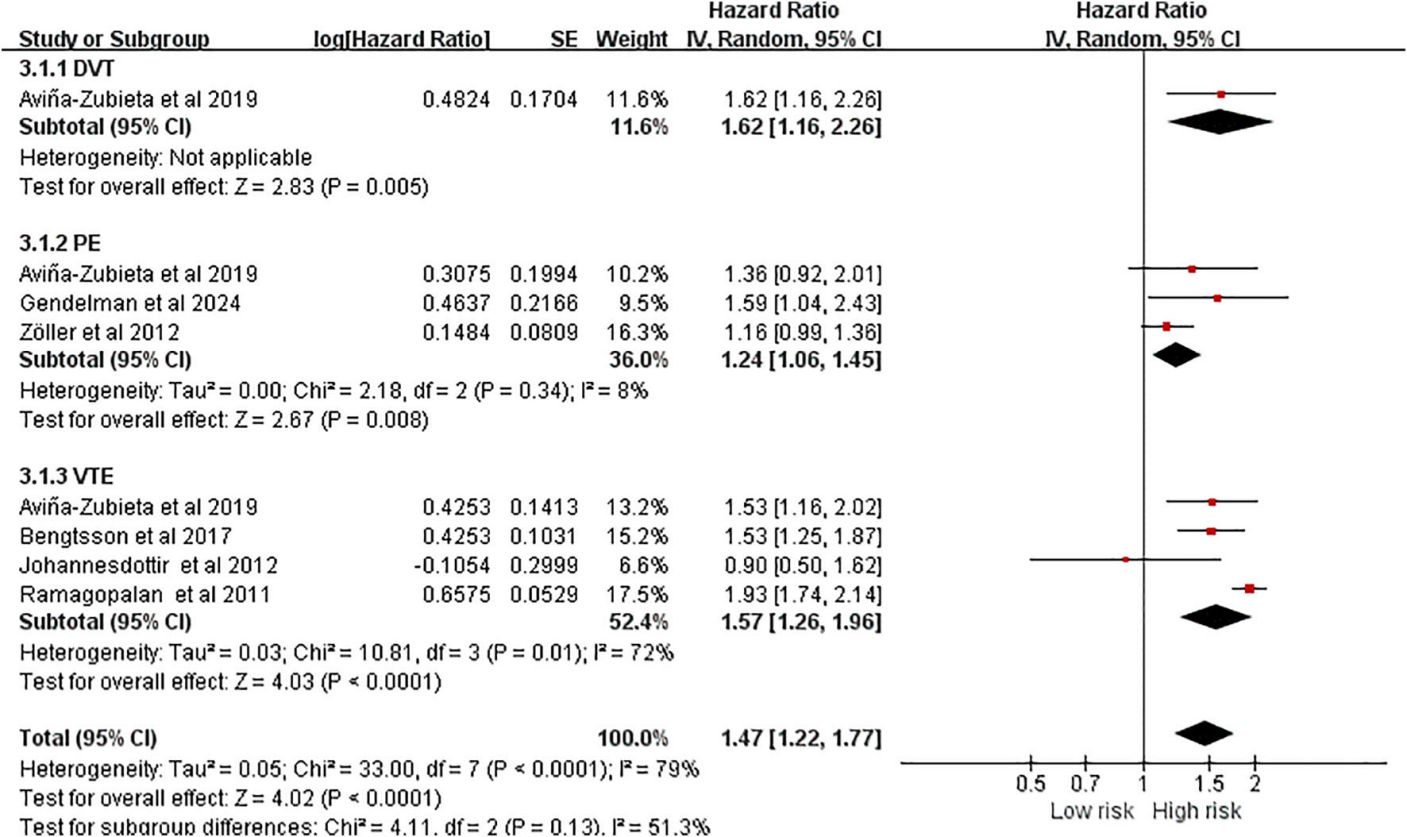
Subgroup analysis forest plot of the risk of venous thromboembolism in ankylosing spondylitis.

Subgroup analysis by thrombosis type was performed to explore heterogeneity and to assess whether the risk association was consistent across the different clinical manifestations of VTE. The results indicated that AS was associated with an elevated risk of all VTE events (VTE: HR = 1.57, 95%CI(1.26, 1.96), *P* < 0.0001; DVT: HR = 1.62, 95%CI(1.16, 2.26), *P* = 0.005; PE: HR = 1.24, 95%CI(1.06, 1.45), *P* = 0.008) (**Figure 3**).

### 3.5 Sensitivity analysis

**Figure 4:**
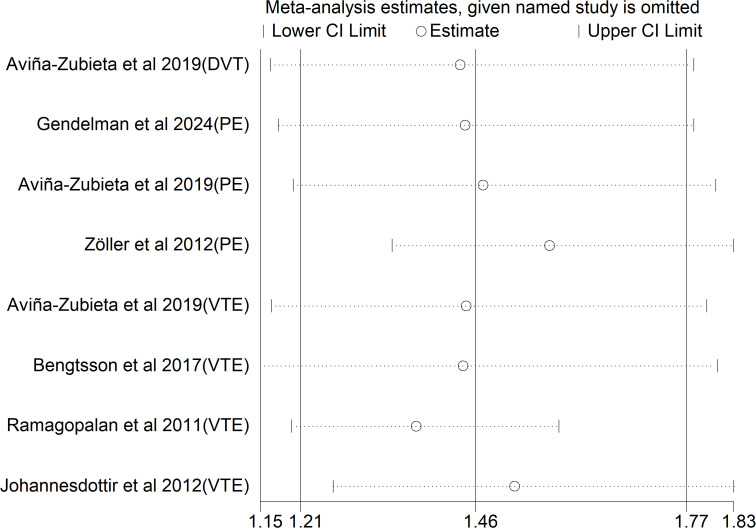
Sensitivity analysis of the risk of venous thromboembolism in ankylosing spondylitis.

A leave-one-out sensitivity analysis showed that the pooled effect size did not change substantially upon sequential removal of each study, supporting the robustness of the results (**Figure 4**).

### 3.6 Publication bias

**Figure 5:**
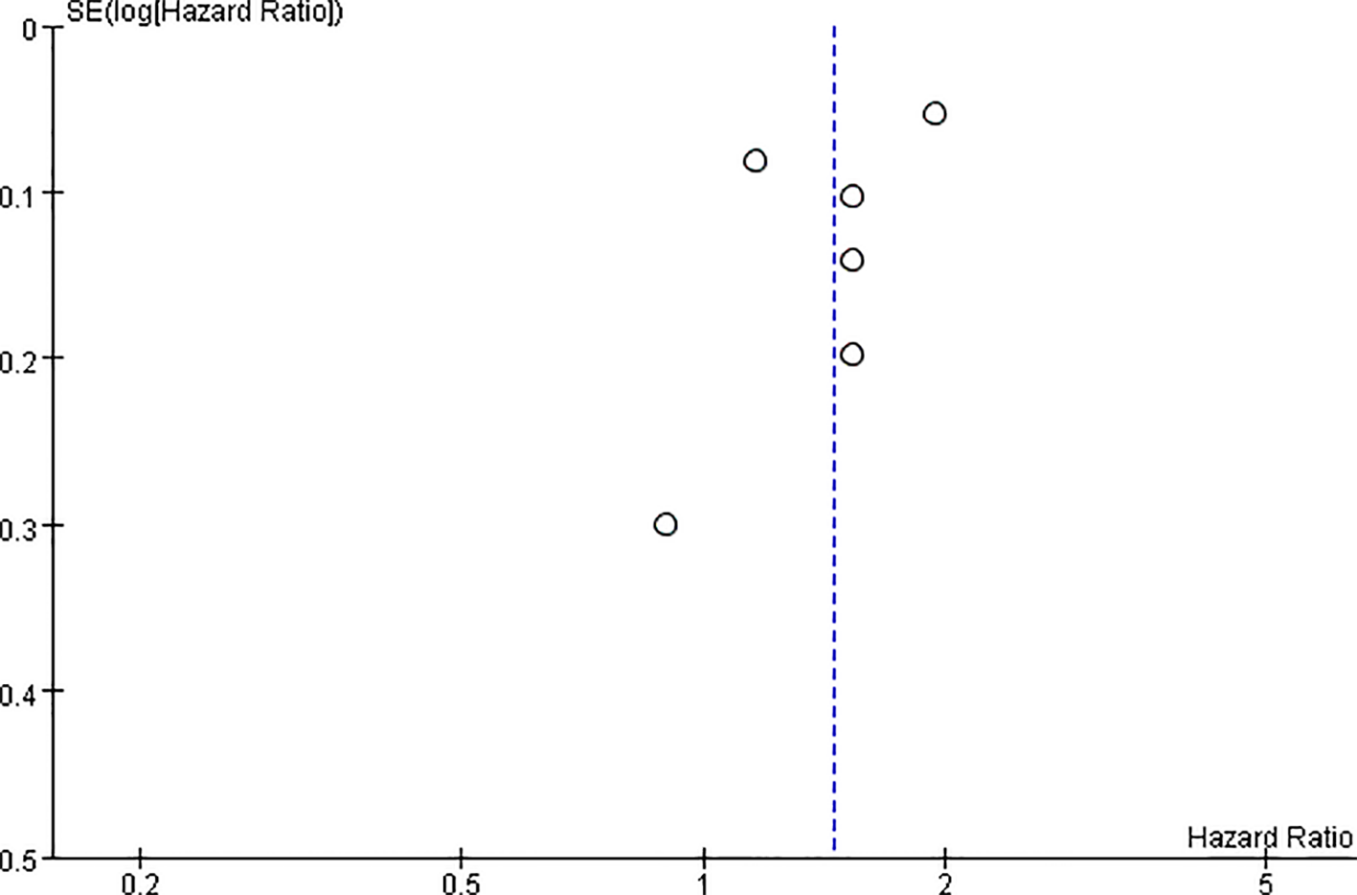
Funnel plot of the risk of venous thromboembolism in ankylosing spondylitis.

Funnel plot inspection suggested approximate symmetry, indicating a low likelihood of publication bias (**Figure 5**). However, due to the limited number of studies (n = 6), the power of this assessment was low, and potential bias cannot be fully ruled out.

### 3.7 GRADE evidence quality assessment

Using the GRADE approach, the initial evidence quality was rated as low because all data came from observational studies. Due to the moderate risk of bias in most studies and substantial heterogeneity, the evidence was downgraded by two levels, resulting in an overall rating of very low (**Supplementary Table S3**).

## 4 Discussion

Numerous studies have indicated an elevated an risk of VTE in patients with various immune-mediated inflammatory diseases (26). This meta-analysis further supports that association by demonstrating a significantly higher risk of VTE in patients with AS. Compared with earlier meta-analyses (27) on this topic, the present study incorporated three recent large-scale, high-quality cohort studies and performed subgroup analyses for VTE, DVT, and PE separately, thereby providing a more detailed and updated assessment of the AS–VTE relationship.

VTE arises from multiple risk factors often conceptualized through Virchow’s triad, including family history, sedentary lifestyle, surgery, trauma, malignancy, pregnancy, oral contraceptive or hormone replacement use, obesity, male sex, smoking, advanced age, anatomical abnormalities, chronic diseases, antiphospholipid syndrome, and genetic predispositions (28, 29). The exact mechanism by which AS contributes to VTE remains unclear. AS is frequently associated with other immune-mediated conditions such as systemic lupus erythematosus, Sjögren’s syndrome, rheumatoid arthritis, antiphospholipid syndrome, Behcet’s disease, and inflammatory bowel disease (30–32). It is well established that several of these disorders, including systemic lupus erythematosus, antiphospholipid syndrome, and inflammatory bowel disease, confer a high risk of VTE (33–35). Behçet’s disease, for instance, commonly presents with DVT and superficial phlebitis (36). Whether the increased VTE risk in AS is independent of these comorbidities remains uncertain. Although Avina-Zubieta et al. (9) reported a significant difference between AS patients with inflammatory bowel disease and controls, no direct comparative analysis of the risk of VTE was provided. Similarly, while Johannesdottir et al. (6) indicated elevated VTE risk in patients with mixed connective tissue disease, no subgroup analysis was performed for AS patients with overlapping immune disorders.

AS is a chronic inflammatory condition driven by upregulation of pro-inflammatory cytokines such as tumor necrosis factor-α (TNF-α), interleukin-1 (IL-1), and IL-23/IL-17 (37, 38). Inflammation and thrombosis are closely interrelated: inflammation can promote thrombus formation, and thrombosis can exacerbate inflammation (39, 40). Recent evidence highlights crosstalk between innate immunity and coagulation cascade activation as a key mechanism in venous thrombogenesis (41). Endothelial activation, Weibel–Palade body release, hypoxia, reactive oxygen species, inflammasome signaling, neutrophil extracellular traps, and other immune mediators collectively contribute to VTE development (42). Notably, AS pathogenesis involves the NLRP3 inflammasome axis, including NLRP3, caspase-1, ASC, IL-1β, IL-17A and IL-23 (43). Targeting the NLRP3 inflammasome may thus represent a promising therapeutic strategy for both AS and AS-related VTE. Furthermore, Goulielmos et al. (44) identified shared genetic factors between AS and VTE, suggesting a common genetic background. The pathogenesis of VTE in AS likely involves a complex interplay of genetic, inflammatory, immune, coagulation, and complement pathways. Elucidating common mechanisms may help overcome therapeutic challenges in both conditions.

This meta-analysis has several limitations. Firstly, since all the included studies were observational studies, they could only indicate correlations but not infer causal relationships. Although adjusted effect estimates were pooled, residual confounding may persist due to variability in adjusted covariates across studies. Second, significant heterogeneity was observed, which may reflect differences in study design, population characteristics, follow-up duration, and confounding adjustment methods. Furthermore, while meta-regression could help explore the observed heterogeneity, the limited number of included studies precluded a meaningful analysis of this kind. Finally, the restriction to English-language publications may introduce language bias. These limitations warrant cautious interpretation of the results.

## 5 Conclusion

This meta-analysis, based on six high-quality observational studies, indicated that AS may be associated with an elevated risk of VTE and its subtypes, including DVT and PE. These findings underscore the importance of clinical vigilance regarding thrombotic risk in patients with AS, particularly when accompanied by traditional risk factors. Future large-scale prospective studies are warranted to validate and quantify the independent association between AS and VTE, elucidate the underlying mechanisms, especially the interplay among inflammation, immune activation, and the coagulation pathways, and identify high-risk patient subgroups to inform targeted preventive strategies.

## Data Availability

The original contributions presented in the study are included in the article/**Supplementary Material**. Further inquiries can be directed to the corresponding author/s.

## Supplementary material

Supplementary Figure 1 Summary of risk of bias assessment using the ROBINS-I tool.

Supplementary Table 1 Search strategy.

Supplementary Table 2 Summary of adjusted confounding variables included in the studies.

Supplementary Table 3 GRADE for quality of evidence profile.
