# Supplementary material for: The association between ankylosing spondylitis and the risk of venous thromboembolism: a meta-analysis
Source: Front Immunol. 2025 Dec 9;16:1670965. doi: 10.3389/fimmu.2025.1670965 (PMC12722833; doi:10.3389/fimmu.2025.1670965)
Supplement: Supplementary Figure 1 — Summary of risk of bias assessment using the ROBINS-I tool. [file Table1.docx]

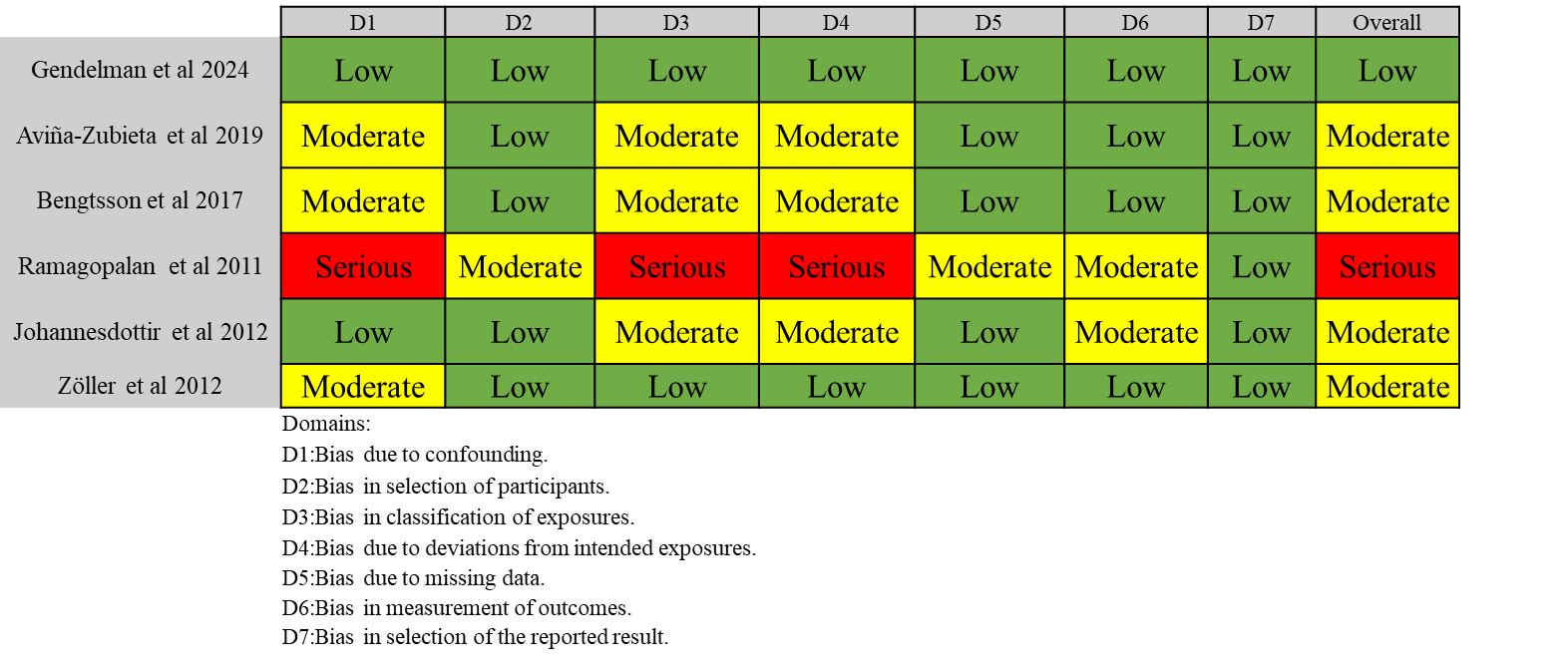


**Supplementary Materials Figure S1.Summary of risk of bias assessment using the ROBINS-I tool.**

| **Supplementary Materials Table S1.Search strategy** | | | | |
| --- | --- | --- | --- | --- |
| Database | No. | Search strategy | Results | Time |
| Embase | #1 | 'vein thrombosis'/exp OR 'vein thrombosis' | 196,648 | June 24,2025 |
|  | #2 | 'phlebothrombos*':ti,ab,kw OR 'venous thrombos*':ti,ab,kw OR 'deep vein thrombos*':ti,ab,kw OR 'deep venous thrombos*':ti,ab,kw | 88,835 |  |
|  | #3 | 'pulmonary embolism*':ti,ab,kw OR 'pulmonary thromboembolism*':ti,ab,kw | 84,938 |  |
|  | #4 | 'lung embolism'/exp | 141,893 |  |
|  | #5 | 'ankylosing spondylitis'/exp | 37,706 |  |
|  | #6 | 'bechterew* disease':ti,ab,kw OR 'marie struempell disease':ti,ab,kw OR 'spondyl* ankylopoietica':ti,ab,kw OR 'rheumatoid spondylitis':ti,ab,kw OR 'ankylosing spondyl*':ti,ab,kw | 32,636 |  |
|  | #7 | #1 OR #2 OR #3 OR #4 | 301,301 |  |
|  | #8 | #5 OR #6 | 43,540 |  |
|  | #9 | #7 AND #8 | 459 |  |
| PubMed | #1 | (((((("Venous Thrombosis"[Mesh]) OR (Phlebothrombos*[Title/Abstract])) OR (Venous Thrombos*[Title/Abstract])) OR (Deep Vein Thrombos*[Title/Abstract])) OR (Deep Venous Thrombos*[Title/Abstract])) OR ((("Pulmonary Embolism"[Mesh]) OR (Pulmonary Embolism*[Title/Abstract])) OR (Pulmonary Thromboembolism*[Title/Abstract]))) AND (((((("Spondylitis, Ankylosing"[Mesh]) OR (Bechterew* Disease[Title/Abstract])) OR (Marie Struempell Disease[Title/Abstract])) OR (Spondyl* Ankylopoietica[Title/Abstract])) OR (Rheumatoid Spondylitis[Title/Abstract])) OR (Ankylosing Spondyl*[Title/Abstract])) | 86 | June 24,2025 |
|  |  | Read the references of the relevant literature. | 8 | June 24,2025 |
| Cochrane | #1 | MeSH descriptor: [Venous Thrombosis] explode all trees | 2,023 | June 24,2025 |
|  | #2 | (Venous Thrombosis):ti,ab,kw OR (Phlebothrombos*):ti,ab,kw OR (Venous Thrombos*):ti,ab,kw OR (Deep Vein Thrombos*):ti,ab,kw OR (Deep Venous Thrombos*):ti,ab,kw | 10,757 |  |
|  | #3 | MeSH descriptor: [Pulmonary Embolism] explode all trees | 1,526 |  |
|  | #4 | (Pulmonary Embolism*):ti,ab,kw OR (Pulmonary Thromboembolism*):ti,ab,kw (Word variations have been searched) | 5,494 |  |
|  | #5 | MeSH descriptor: [Spondylitis, Ankylosing] explode all trees | 944 |  |
|  | #6 | (Bechterew* Disease):ti,ab,kw OR (Marie Struempell Disease):ti,ab,kw OR (Spondyl* Ankylopoietica):ti,ab,kw OR (Rheumatoid Spondylitis):ti,ab,kw OR (Ankylosing Spondyl*):ti,ab,kw | 2,953 |  |
|  | #7 | #1 or #2 or #3 or #4 | 14,227 |  |
|  | #8 | #5 or #6 | 2,953 |  |
|  | #9 | #7 and #8 | 14 |  |
| Web of Science | #1 | (((((((TS=(Venous Thrombosis)) OR TS=(Phlebothrombos*)) OR TS=(Venous Thrombos*)) OR TS=(Deep Vein Thrombos*)) OR TS=(Deep Venous Thrombos*)) OR TS=(Pulmonary Embolism)) OR TS=(Pulmonary Embolism*)) OR TS=(Pulmonary Thromboembolism*) and Preprint Citation Index (Exclude – Database) | 90,619 | June 24,2025 |
|  | #2 | (((((TS=(Spondylitis, Ankylosing)) OR TS=(Bechterew* Disease)) OR TS=(Marie Struempell Disease)) OR TS=(Spondyl* Ankylopoietica)) OR TS=(Rheumatoid Spondylitis)) OR TS=(Ankylosing Spondyl*) and Preprint Citation Index (Exclude – Database) | 20,960 |  |
|  | #3 | #1 AND #2 and Preprint Citation Index (Exclude – Database) | 54 |  |

| **Supplementary Materials Table S2. Summary of adjusted confounding variables included in the studies..** | | | | | | | | | |  |
| --- | --- | --- | --- | --- | --- | --- | --- | --- | --- | --- |
| Study | Age | Sex | BMI | Ethnicity | Comorbidity | Drugs | Smoking history | Trauma/surgical history | Economic status | Number of outpatient visits |
| Gendelman et al 2024 | √ | √ | √ | √ | × | × | √ | × | √ | × |
| Aviña-Zubieta et al 2019 | √ | √ | × | × | × | √ | × | × | × | √ |
| Bengtsson et al 2017 | √ | √ | × | × | × | × | × | × | × | × |
| Ramagopalan et al 2011 | √ | √ | × | × | × | × | × | × | √ | × |
| Johannesdottir et al 2012 | √ | √ | × | × | √ | √ | × | √ | √ | × |
| Zöller et al 2012 | √ | √ | × | × | √ | × | × | × | × | × |

| **Supplementary Materials Table S3. GRADE for quality of evidence profile.** | | | | | | | | |
| --- | --- | --- | --- | --- | --- | --- | --- | --- |
| Outcomes | Number of studies(patients) | Risk of bias | Inconsistency | Indirectness | Inprecision | Other considerations | HR(95% CI) | Certainty |
| VTE | 6(601585) | Serious^a^ | Serious^b^ | Not Serious | Not Serious | None | 1.47 (1.22, 1.77) | ⊕⊝⊝⊝  Very low |
| Note:a,the score was downgraded because most studies have a moderate risk of bias; b,the score was downgraded because subatantial heterogeneity between studies was detected;VTE, venous thromboembolism;HR,hazard ratio;CI,confidence interval;GRADE,Grading of Recommendations Assessment, Development and Evaluations. | | | | | | | | |
